# Ecofriendly Synthesis of Silver Nanoparticles by *Terrabacter humi* sp. nov. and Their Antibacterial Application against Antibiotic-Resistant Pathogens

**DOI:** 10.3390/ijms21249746

**Published:** 2020-12-21

**Authors:** Shahina Akter, Sun-Young Lee, Muhammad Zubair Siddiqi, Sri Renukadevi Balusamy, Md. Ashrafudoulla, Esrat Jahan Rupa, Md. Amdadul Huq

**Affiliations:** 1 Department of Food Science and Biotechnology, Gachon University, Seongnam 461-701, Korea; shahinabristy16@gmail.com; 2 Department of Food and Nutrition, Chung-Ang University, Anseong 17546, Korea; nina6026@cau.ac.kr; 3 Department of Biotechnology, Hankyong National University, Anseong 17546, Korea; mzsiddiqi1988@yahoo.com; 4 Department of Food Science and Technology, Sejong University, Seoul 143-747, Korea; renucoimbatore@gmail.com; 5 Department of Food Science and Technology, Chung-Ang University, Anseong 17546, Korea; ashrafmiu584@gmail.com; 6 Department of Oriental Medicinal Material Processing, Kyung Hee University, Yongin 17104, Korea; eshratrupa91@gmail.com

**Keywords:** *Terrabacter humi* MAHUQ-38^T^, AgNPs, eco-friendly synthesis, multi-drug-resistant microorganisms

## Abstract

It is essential to develop and discover alternative eco-friendly antibacterial agents due to the emergence of multi-drug-resistant microorganisms. In this study, we isolated and characterized a novel bacterium named *Terrabacter humi* MAHUQ-38^T^, utilized for the eco-friendly synthesis of silver nanoparticles (AgNPs) and the synthesized AgNPs were used to control multi-drug-resistant microorganisms. The novel strain was Gram stain positive, strictly aerobic, milky white colored, rod shaped and non-motile. The optimal growth temperature, pH and NaCl concentration were 30 °C, 6.5 and 0%, respectively. Based on 16S rRNA gene sequence, strain MAHUQ-38^T^ belongs to the genus *Terrabacter* and is most closely related to several *Terrabacter* type strains (98.2%–98.8%). *Terrabacter humi* MAHUQ-38^T^ had a genome of 5,156,829 bp long (19 contigs) with 4555 protein-coding genes, 48 tRNA and 5 rRNA genes. The culture supernatant of strain MAHUQ-38^T^ was used for the eco-friendly and facile synthesis of AgNPs. The transmission electron microscopy (TEM) image showed the spherical shape of AgNPs with a size of 6 to 24 nm, and the Fourier transform infrared (FTIR) analysis revealed the functional groups responsible for the synthesis of AgNPs. The synthesized AgNPs exhibited strong anti-bacterial activity against multi-drug-resistant pathogens, *Escherichia coli* and *Pseudomonas aeruginosa*. Minimal inhibitory/bactericidal concentrations against *E. coli* and *P. aeruginosa* were 6.25/50 and 12.5/50 μg/mL, respectively. The AgNPs altered the cell morphology and damaged the cell membrane of pathogens. This study encourages the use of *Terrabacter humi* for the ecofriendly synthesis of AgNPs to control multi-drug-resistant microorganisms.

## 1. Introduction

The genus *Terrabacter* was first proposed by Collins et al. [1] through the transfer of *Pimelobacter tumescens* to *Terrabacter tumescens*. The *Terrabacter* genus comprises 10 species with valid published names at the time of writing (https://lpsn.dsmz.de/genus/terrabacter). Species of the genus are characterized as rod or coccus shape, Gram-positive, catalase-positive, obligately aerobic bacteria and have C_14:0 iso_ and C_15:0 iso_ as the major fatty acids and MK- 8(H4) as the predominant respiratory quinone. The members of this genus have DNA G + C content from 70 to 73 mol% [2,3]. The members of this genus have been isolated from different sources including soil, air, stone, flowerbeds, etc. [1,2,3,4,5,6]. In the present study, we isolated a novel species of the genus *Terrabacter*, *Terrabacter humi* sp. nov., designated as MAHUQ-38^T^, isolated from the soil sample of a grape garden.

Nanotechnology is broadly classified into different sectors, including chemical, physical and biogenic [7]. Among these three methods, researchers are more interested in biogenic methods because of their various advantages over the other processes [8]. Both physical and chemical methods have many drawbacks, including the use of toxic chemicals, synthesis of harmful byproducts, they require enormous energy and pressure, etc. [9,10]. The biogenic method of nanoparticle (NP) synthesis is not only ecofriendly but also the biosynthesized NPs have many good properties, such as optical, chemical and photoelectrical properties that make them diverse for industrial applications [11]. The ecofriendly synthesized NPs have different biomedical applications, such as antibacterial agents, magnetic resonance imaging, carriers for targeted drug delivery, gene therapy, biosensors, cancer treatment, etc. [12].

For the ecofriendly synthesis of NPs, a number of microorganisms such as bacteria, algae, fungi, etc., have been isolated from various sources, including foods, soil, plants, etc. [13,14,15,16]. Among all of these microorganisms, bacteria-mediated NP synthesis is the favored one, because of their easy genetic manipulation and handling, such as, *Paenibacillus anseongense*, *Bacillus subtilis*, *Bacillus methylotrophicus*, *Sporosarcina koreensis*, and *Microvirga rosea* [10,12,13,14,17]. Ecofriendly synthesis of various metal NPs has gained considerable interest in different sectors of research and development. Among various metal NPs, silver nanoparticles (AgNPs) have achieved considerable attention, due to their strong antimicrobial, anticancer, catalytic, optical and electrical properties [14,18]. AgNPs are being used as an antimicrobial agent to control pathogens, an anticancer agent to treat different types of cancers, probes for bioimaging, an anti-inflammatory agent and targeted drug delivery system [14,16,18]. AgNPs are also being considered for the utilization in numerous industrial products including hand gel, wound dressings, medical catheter coverings, footwear, etc., due to their strong antimicrobial properties [18,19].

The emergence of multi-drug-resistant microorganisms is a serious concern for human health. So, it is essential to find out the alternative antimicrobial agent to control the multi-drug-resistant microorganisms. Ecofriendly synthesized AgNPs could be a potential candidate in this regard. The present study is an attempt of isolation and characterization of novel bacterial species *T. humi* MAHUQ-38^T^ and utilization of this novel species for ecofriendly, facile synthesis of AgNPs and further subjected to characterization and evaluation of antibacterial efficacy against multi-drug-resistant human pathogens.

## 2. Results and Discussion

### 2.1. Phenotypic and Biochemical Characteristics

**Figure 1:**
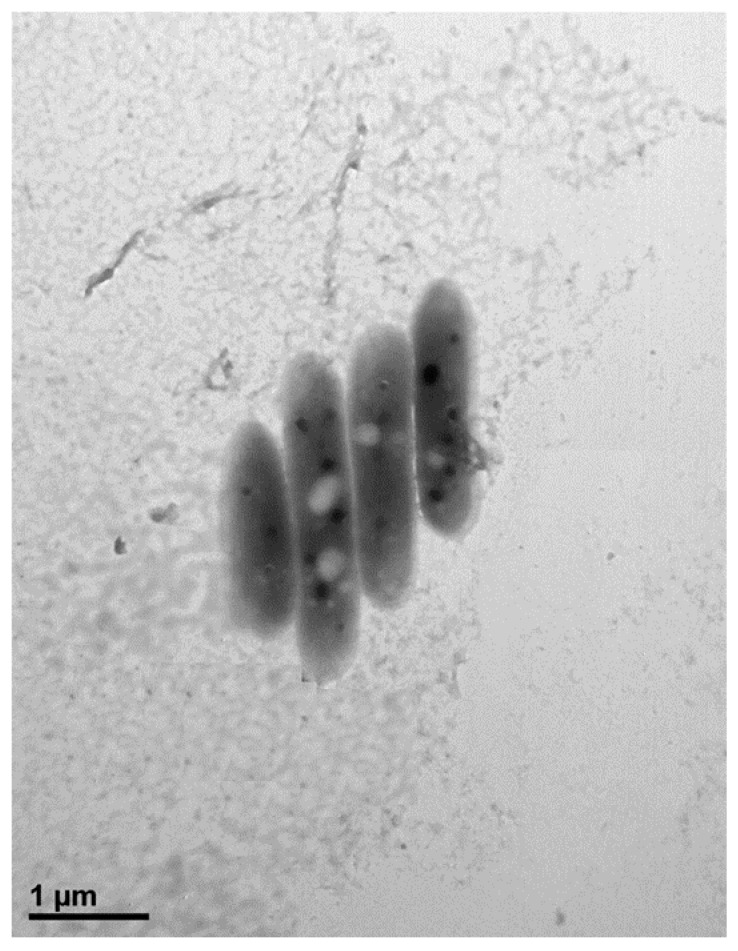
Transmission electron micrograph of cells of *Terrabacter humi* MAHUQ-38^T^ after negative staining with uranyl acetate, Bar, 1.0 μm.

Cells of strain MAHUQ-38^T^ were Gram positive, strictly aerobic, non-motile, and rod shaped with 0.3–0.7 μm wide and 1.6–2.9 μm long (Figure 1). Colonies of MAHUQ-38^T^ grown on Reasoner’s 2A (R2A) agar medium were spherical, milky white colored and 0.1–0.5 mm in diameter after 48 h of incubation at 30 °C. The isolate can grow on R2A agar, Luria–Bertani (LB) agar, nutrient agar (NA) and tryptic soy agar (TSA), but did not grow on MacConkey agar. The strain was positive for catalase, but negative for oxidase. They are able to hydrolysis of casein, DNA, l-tyrosine, esculin, gelatin, Tween 80 and Tween 20, but negative for starch and urea. Phenotypic examination showed that strain MAHUQ-38^T^ shared several traits in common with its close relatives. However, there are some morphological, physiological and biochemical differences between strain MAHUQ-38^T^ and phylogenetically related *Terrabacter* species that differentiate this novel strain from related type strains (Table 1). Strain MAHUQ-38^T^ could be clearly differentiated from the phylogenetically most closely relative, *T. tumescens* DSM 20308^T^ by the ability to hydrolyse *a*-glucosidase; the presence of alkaline phosphatase, valine arylamidase, cystine arylamidase, *a*-glucosidase and *a*-galactosidase activities. Physiological characteristics of strain MAHUQ-38^T^ are summarized in the species description and in Table 1.

### 2.2. 16S rRNA Gene Sequence and Phylogenetic Analysis

**Figure 2:**
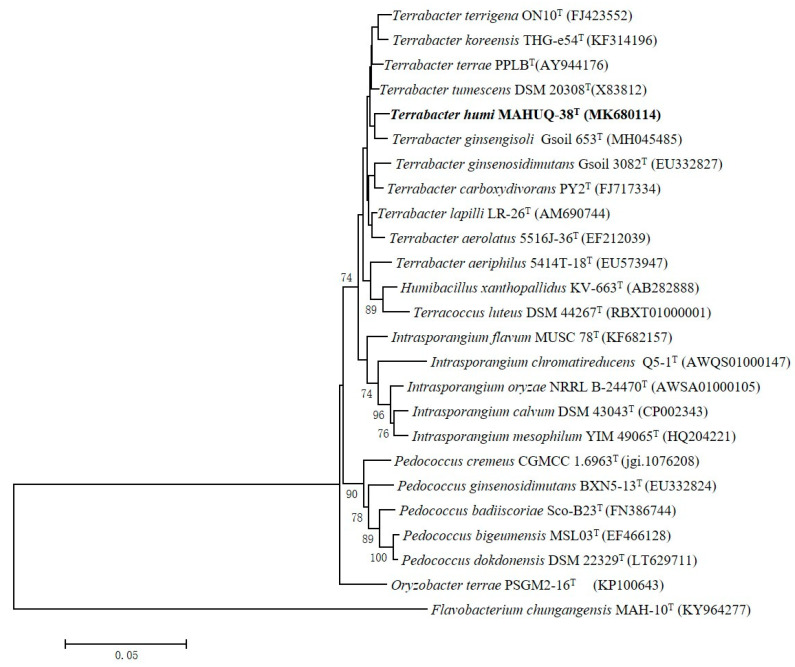
Neighbor-joining (NJ) tree based on 16S rRNA gene sequence analysis showing phylogenetic relationships of strain MAHUQ-38^T^ and members of genus *Terrabacter*. Bootstrap values more than 70% based on 1000 replications are shown at branching points. Scale bar, 0.05 substitutions per nucleotide position.

A 1456 nt long 16S rRNA gene sequence of strain MAHUQ-38^T^ was identified and, subsequently, this sequence was used for comparative analysis (GenBank accession number, MK680114). 16S rRNA gene sequence similarity calculations indicated that strain MAHUQ-38^T^ belongs to the genus *Terrabacter* and was closely related to *T. tumescens* DSM 20308^T^ (98.8%), *T. terrae* PPLB^T^ (98.6%), *T. terrigena* ON10^T^ (98.5%), *T. lapilli* LR-26^T^ (98.4%), *T. ginsengihumi* Gsoil 653^T^ (98.4%) and *T. aerolatus* 5516J-36^T^ (98.3%). Phylogenetic analysis using the neighbor-joining method on the basis of 16S rRNA gene sequences also showed that the strain MAHUQ-38^T^ was clustered within the genus *Terrabacter* (Figure 2).

### 2.3. Draft Genome and DNA G + C Content Analysis

The draft genome of strain MAHUQ-38^T^ contains 19 contigs with an N50 size of 910,522 bp. The total genome size was 5,156,829 bp, with an average G + C content of 70.8 mol%. Gene prediction and annotation by PGAP resulted in the identification of 4555 protein-encoding genes, 5 rRNA genes and 48 tRNA genes. The NCBI accession number for the genome sequence of strain MAHUQ-38^T^ is JACVCU000000000. Genome sequence features of strain MAHUQ-38^T^ are shown in Table 2. On the other hand, the closest type strain, *T. tumescens* JCM 1365^T^, contains a 4,707,860 bp long genome with an N50 size of 611,474 bp, 71.0 mol% GC, 4295 protein-encoding genes, three rRNA genes and 46 tRNA genes (http://gctype.wdcm.org/sequencing/GCM10009721). The genomic ANI (average nucleotide identity) values between strain MAHUQ-38^T^ and the most close type strains *T. tumescens* JCM 1365^T^ and *T. aerolatus* 5516J-36^T^ were 83.7% and 83.4%, respectively (Table S1), which were well below (≥95–96%) to suggest a novel species. The digital DNA-DNA hybridization (dDDH) value based on the draft genomes between strain MAHUQ-38^T^ and close relatives *T. tumescens* JCM 1365^T^ and *T. aerolatus* 5516J-36^T^ were 27.2% and 26.7%, respectively (Table S1), which were far below the threshold value (70%) for species delineation.

### 2.4. DNA-DNA Hybridization

MAHUQ-38^T^ showed DNA-DNA relatedness values of 38.9 ± 1.3% with *T. tumescens* KACC 20535^T^, 36.6 ± 1.0% with *T. terrae* KACC 11642^T^ and 34.36 ± 1.2% with *T. terrigena* KCTC 19602^T^. Since the DNA-DNA relatedness values were much lower, strain MAHUQ-38^T^ was determined as a novel species of the genus *Terrabacter* [20].

### 2.5. Cellular Fatty Acid, Respiratory Quinones and Polar Lipid Analysis

The major fatty acids of MAHUQ-38^T^ were C_14:0 iso_ (24.6%), C_15:0 iso_ (24.4%), C_16:0 iso_ (16.8%) and C_15:0 anteiso_ (12.3%). Some quantitative and qualitative differences in the fatty acid profiles of close type strains that distinguished strain MAHUQ-38^T^ from close type strains of genus *Terrabacter* are shown in Table 3. The major respiratory quinone of strain MAHUQ-38^T^ was menaquinone MK-8 (H4) which is one of the common characteristics of genus *Terrabacter* [1,3]. Polar lipids of strain MAHUQ-38^T^ comprised diphosphatidylglycerol, phosphatidylethanolamine, phosphatidylglycerol, phosphatidylinositol and some unknown lipids (Figure S1). The similar polar lipid profiles were detected in the close relatives of the *Terrabacter* genus [2,3,4].

### 2.6. Taxonomic Conclusion

The phenotypic, chemotaxonomic and biochemical features and the phylogenetic inference support the proposition that strain MAHUQ-38^T^ represents a novel species of the genus *Terrabacter*, for which the name *Terrabacter humi* sp. nov. is proposed.

### 2.7. Description of Terrabacter humi sp. nov.

#### *Terrabacter humi* (hu’mi. L. gen. n. *humi* of/from soil)

Cells are strictly aerobic, Gram stain positive, non-motile and rod shaped and are 0.3–0.7 µm wide and 0.9–2.0 µm long. Cells grow well on R2A agar, LB agar, NA and TSA, but did not grow on MacConkey agar. Colonies on R2A agar are milky white and spherical in shape. The colony size is 0.1–5.0 mm after 48 h of growth on R2A agar. Growth occurs at 10–40 °C (optimum, 30 °C), at pH 4.5–10.0 (optimum, pH 6.5) and with 0–5% NaCl (optimum, 0%). The cells were positive for the catalase test and hydrolysis of casein, DNA, l-tyrosine, Tween 80 and Tween 20; they were negative for the oxidase test and hydrolysis of starch and urea. Flexirubin-type pigments are absent. In API 20NE tests, the cells were positive for the hydrolysis of aesculin and gelatin, nitrate reduction and β-galactosidase (PNPG) activities, assimilation of mannose, arabinose, glucose, maltose, *N*-acetyl-glucosamine, gluconate, and malate, but they were negative for indole production, d-glucose acidification, urease and arginine dihydrolase activities, and assimilation of mannitol, caprate, adipate, citrate and phenyl-acetate. In the API ZYM tests, valine arylamidase, acid phosphatase, esterase lipase (C8), alkaline phosphatase, C4 esterase, leucine arylamidase, trypsin, cystine arylamidase, naphthol-AS-BI-phosphohydrolase, β-glucosidase, β-galactosidase, α-glucosidase, α-galactosidase and lipase (C14) activities are present, but α-chymotrypsin, α-mannosidase, β-glucuronidase, α-fucosidase and *N*-acetyl-β-glucosaminidase activities are absent. The major fatty acids were C_14:0 iso_, C_15:0 iso_, C_16:0 iso_ and C_15:0 anteiso_. The predominant respiratory quinone was menaquinone MK-8(H4). The main polar lipids were diphosphatidylglycerol, phosphatidylethanolamine, phosphatidylglycerol and phosphatidylinositol. The DNA G + C content of the type strain MAHUQ-38^T^ is 70.8 mol%.

The type strain is MAHUQ-38^T^ (=KACC 21245^T^ = CGMCC 1.17350^T^), isolated from the soil sample of a grape garden, Anseong, South Korea.

### 2.8. Ecofriendly Synthesis of AgNPs Using Terrabacter humi sp. nov.

**Figure 3:**
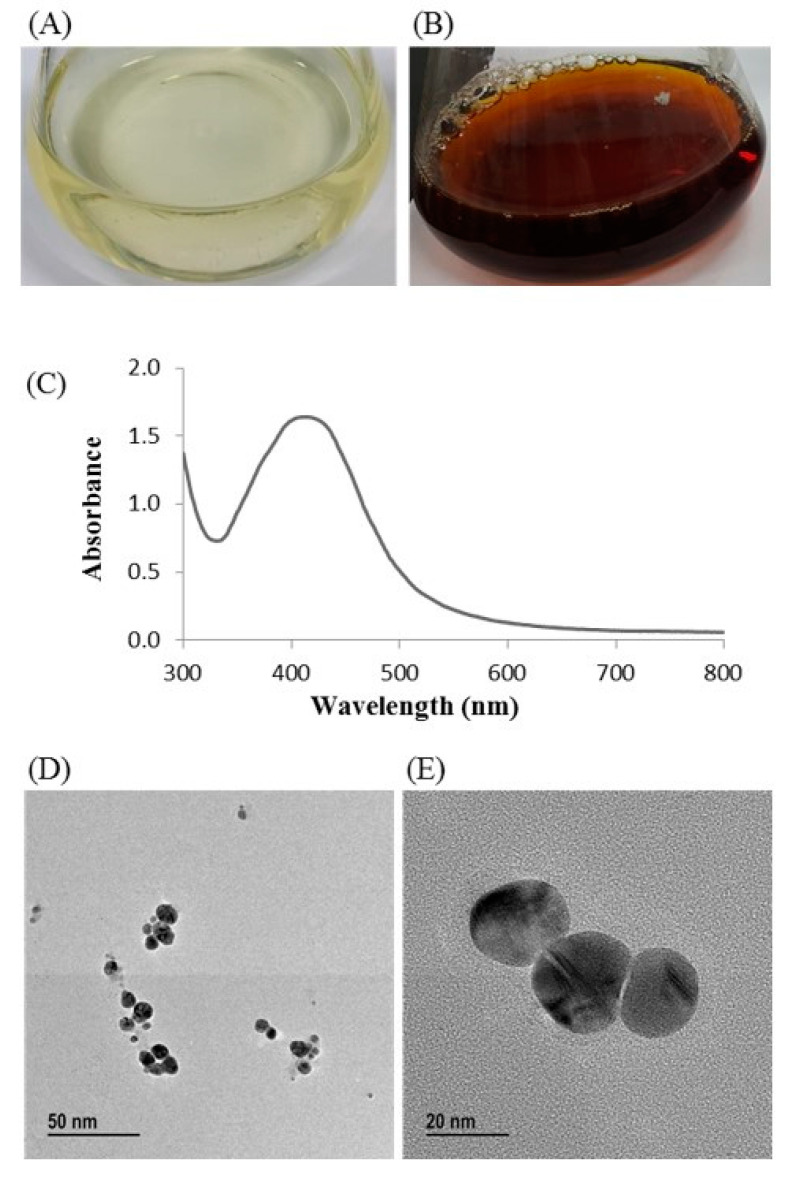
Reasoner’s 2A (R2A) broth with AgNO3 as a control (**A**); ecofriendly synthesized silver nanoparticles (AgNPs) (**B**); ultraviolet–visible (UV–vis) spectra (**C**); and field emission-transmission electron microscopy (FE-TEM) images of ecofriendly synthesized silver nanoparticles (**D**,**E**).

Biosynthesis of silver nanoparticles by *T. humi* MAHUQ-38^T^ was confirmed by visual observation of the color of culture supernatant. The color of MAHUQ-38^T^ culture supernatant was shifted to deep brown from watery yellow within 48 h of incubation (Figure 3B). The color change in culture supernatant indicates the synthesis of silver nanoparticles [13]. In the case of the control, there was no color change (Figure 3A). In the present study, the extracellular methodology was used for the synthesis of AgNPs using *T. humi* MAHUQ-38^T^ which is simple, green, ecofriendly and cost-effective.

### 2.9. Characterization of Synthesized AgNPs

**Figure 4:**
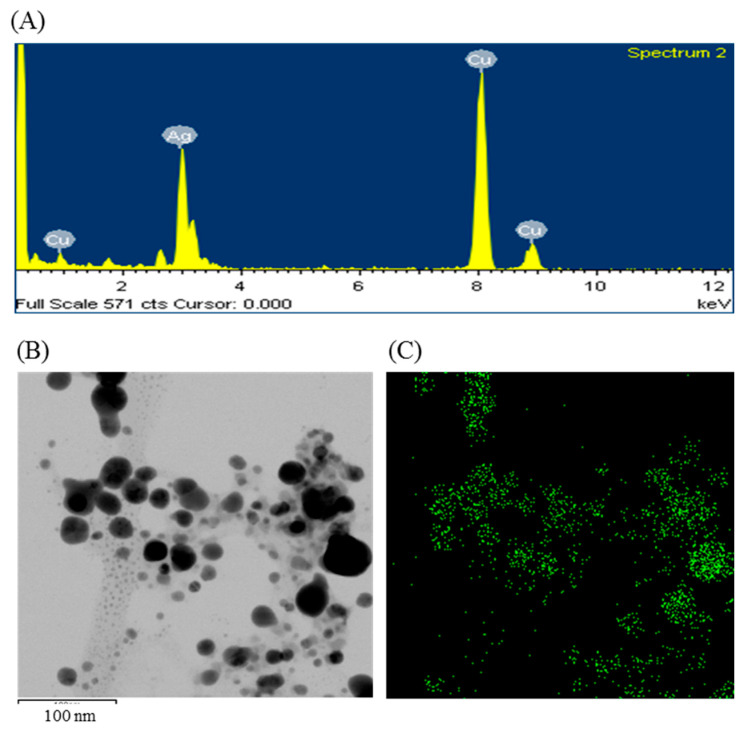
Energy dispersive X-ray (EDX) spectrum of ecofriendly synthesized AgNPs (**A**); TEM image (electron micrograph region) used for mapping (**B**); and the distribution of silver in elemental mapping (**C**).

**Figure 5:**
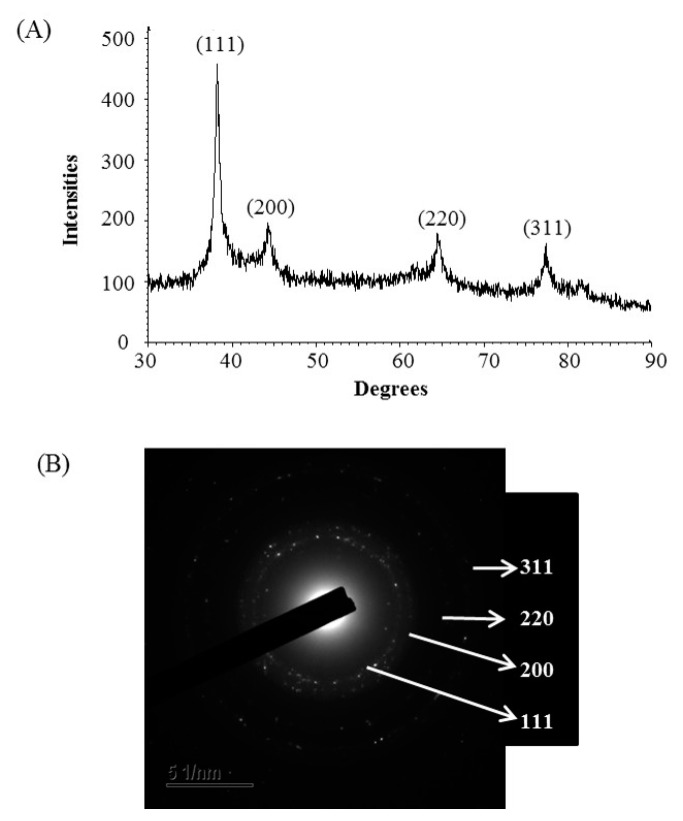
X-ray diffraction pattern (**A**) and selected area diffraction (SAED) pattern (**B**) of ecofriendly synthesized AgNPs.

The formulation of AgNPs was assured by using an ultraviolet–visible (UV–vis) spectral scan at 300–800 nm. A strong peak at around 413 nm was observed (Figure 3C), which confirmed the synthesis of AgNPs [21,22]. FE-TEM (field emission transmission electron microscopy) analysis revealed the spherical shape of eco-friendly synthesized AgNPs and the size ranges from 6 to 24 nm (Figure 3D,E). The EDX (energy dispersive X-ray) spectrum revealed the signal for silver atoms nearly at 3 keV (Figure 4A). Through EDX spectrometer analysis, it was found that the purity of synthesized AgNPs was high (Figure 4A). Some other peaks were also found in the EDX spectrum due to the use of a copper grid in FE-TEM [10]. The elemental mapping results of synthesized silver nanoparticles exhibited the highest distribution of silver elements in the sample (Figure 4B,C). The XRD (X-ray diffraction) analysis revealed several peaks at 2θ values of 38.139°, 44.256°, 64.437° and 77.459° match the respective 111, 200, 220 and 311 lattice planes of AgNPs. These diffraction peaks may be for having the capping agents like biomolecules which are involved for stabilizing the NPs (Figure 5A). The obtained XRD results were similar with a recently reported study which showed the synthesis of silver nanoparticles using microorganisms [14]. The SAED (selected area diffraction) spectrum revealed sharp rings which indicated the crystalline structure of ecofriendly synthesized AgNPs and correlate with the following reflections at 111, 200, 220 and 311 (Figure 5B).

**Figure 6:**
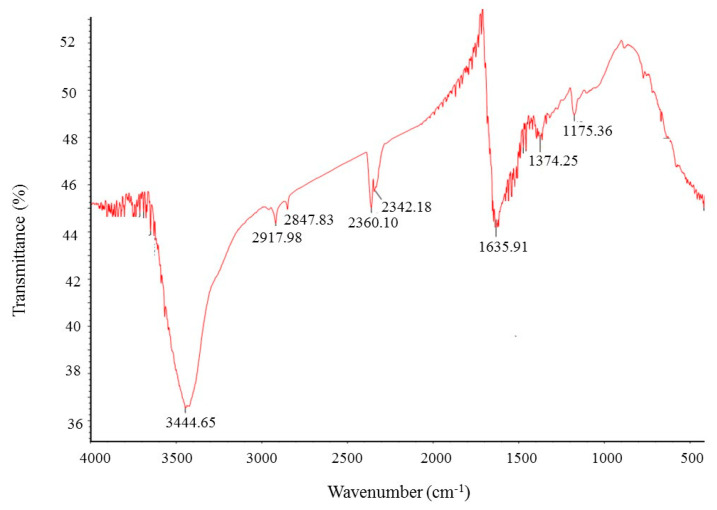
Fourier transform infrared (FT-IR) spectra of ecofriendly synthesized silver nanoparticles.

FT-IR (Fourier transform infrared) analysis was conducted to investigate the bio-active compounds, which may be responsible for the synthesis and stability of AgNPs as a capping agent. The FT-IR pattern showed different peaks throughout the entire range of analysis. FTIR analysis revealed visible bands at 3444.65, 2917.98, 2847.83, 2360.10, 2342.18, 1635.91, 1374.25 and 1175.36 cm^−1^ for biosynthesized silver nanoparticles (Figure 6). The band observed at 3444.65 cm^−1^ indicates O–H (alcohol) and/or N–H (amine) stretching. Bands observed at 2917.98 and 2847.83 cm^−1^ are attributed to C–H (alkane) stretching. The bands seen at 2360.10 and 2342.18 cm^−1^ represent O=C=O (carbonyl bond group). The band at 1635.91 cm^−1^ represents the N–H (amine) group. The bands observed at 1374.25 and 1175.36 cm^−1^ attributed to the C–H (alkane) and C–O (alcohol/ether) group, respectively. The FT-IR analysis revealed the presence of bio-active compounds, which may be responsible for the synthesis and stability of AgNPs. The present study was supported by the previous reports [23,24].

### 2.10. Antimicrobial Activity of Synthesized AgNPs

**Figure 7:**
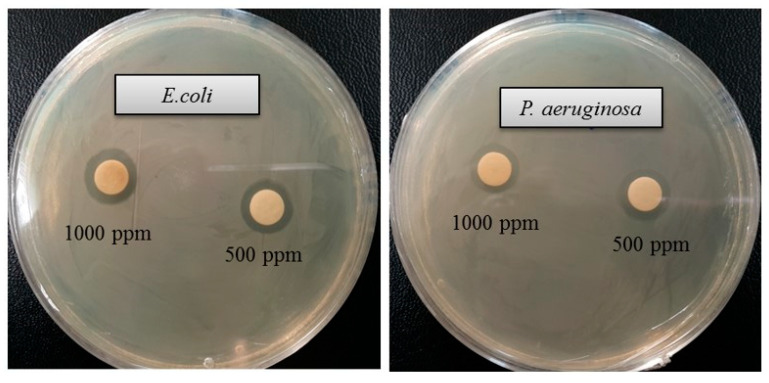
Zones of inhibition of ecofriendly synthesized AgNPs (30 μL) at 500 and 1000 ppm concentrations in water against *E. coli* and *P. aeruginosa.*

**Figure 8:**
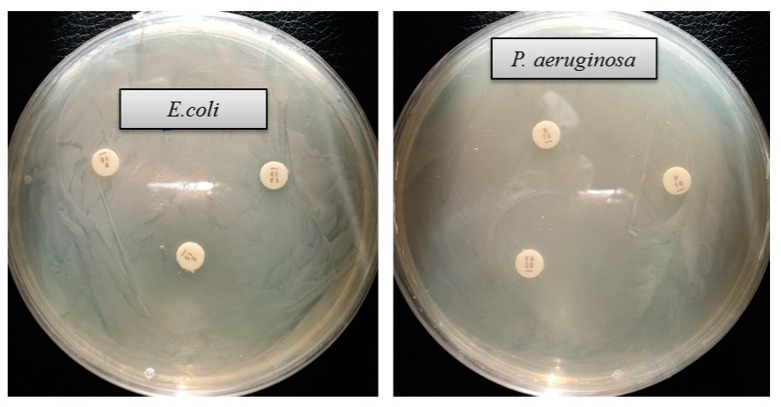
Zones of inhibition of commercial antibiotics against *E. coli and P. aeruginosa*. Abbreviation: VA (vancomycin, 30 μg/disc), E (erythromycin, 15 μg/disc) and P (penicillin, G 10 μg/disc).

The emergence of multi-drug-resistant microorganisms is a serious concern for the medical sector, as it is difficult to control them by available antibiotics. So, it is essential to find out an alternative antimicrobial agent to control these multi-drug-resistant microorganisms. Ecofriendly synthesized AgNPs could be a potential candidate to control them. In the present study, AgNPs were synthesized using green technique and their antibacterial activity was investigated against drug-resistant pathogenic microorganisms, such as *Pseudomonas aeruginosa* and *Escherichia coli*. The results show that eco-friendly synthesized AgNPs have strong antibacterial efficacy against tested pathogens such as *E. coli* and *P. aeruginosa* (Figure 7). Figure 7 reveals the inhibition zones around the paper discs treated with AgNPs. The average results of the zone of inhibition are shown in Table 4. The antibacterial activity against pathogens was measured by calculating the diameter of the inhibition zone. The ecofriendly synthesized AgNPs showed highest antimicrobial activity against antibiotic-resistant *E. coli* followed by *P. aeruginosa*. The results suggest that *Terrabacter humi* MAHUQ-38^T^ mediated AgNPs could be useful as an antimicrobial agent to control drug-resistant pathogens. The results of the present study were consistent with previously reported studies [22,25]. Both pathogens, *P. aeruginosa* and *E. coli*, were resistant to the three tested commercial antibiotics. The commercial antibiotics that were used in this study did not show any activity against tested pathogens (Figure 8, Table 5).

### 2.11. Minimal Inhibitory Concentration (MIC) and Minimal Bactericidal Concentration (MBC)

**Figure 9:**
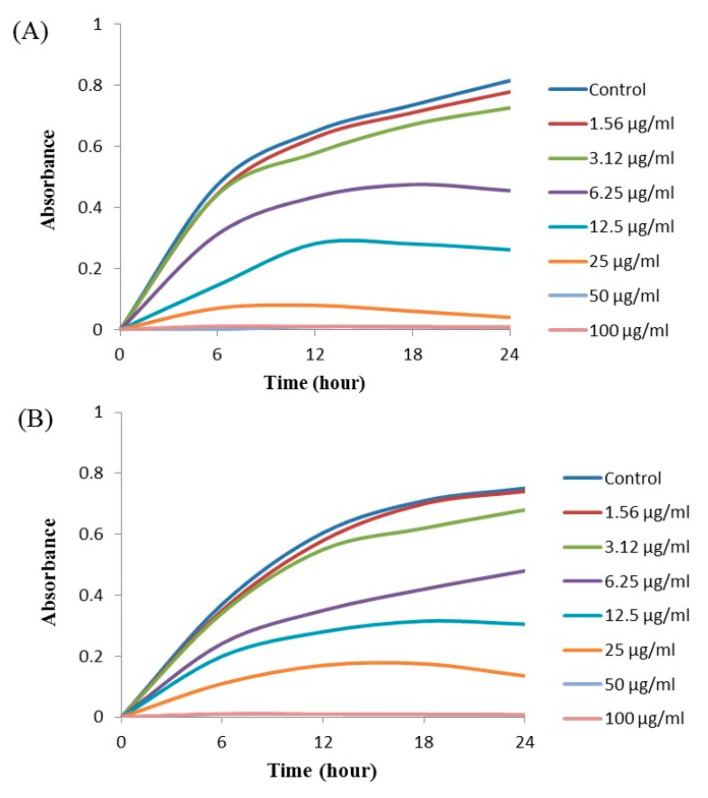
Growth curves of *E. coli* (**A**) and *P. aeruginosa* (**B**) cultured in Mueller–Hinton broth (MHB) with different concentrations of the synthesized AgNPs to determine the Minimal Inhibitory Concentration (MIC).

**Figure 10:**
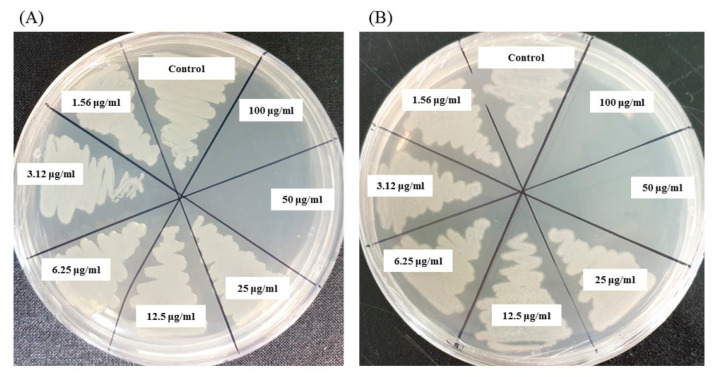
MBC of synthesized AgNPs against *E. coli* (**A**) and *P. aeruginosa* (**B**).

The MIC and MBC of AgNPs against *E. coli* and *P. aeruginosa* were determined by a standard broth microdilution method. From the results, it was noted that the green synthesized AgNPs showed an MIC of 6.25 μg/mL and 12.5 μg/mL for *E. coli* and *P. aeruginosa*, respectively, indicating that the synthesized AgNPs strongly suppressed the proliferation of these pathogens (Figure 9). The MIC of the AgNPs synthesized by strain MAHUQ-38^T^ against *E. coli* were much lower than some other antimicrobial agents, including biosynthesized CuO-NPs and ZnO-NPs which were 0.5 mg/mL and 8 mg/mL, respectively, against *E. coli* [26,27]. The MICs of the AgNPs synthesized by strain MAHUQ-38^T^ against *P. aeruginosa* were also significantly lower than some other antimicrobial agents, including biogenic nanoparticles, which were in the range 0.375–1.6 mg/mL against *P. aeruginosa* [28,29]. The MBCs of biosynthesized AgNPs for both *E. coli* and *P. aeruginosa* were 50 μg/mL (Figure 10), which was also much lower than those of some other antimicrobial agents [26,27,28].

### 2.12. Investigation of Structural Changes by FE-SEM

**Figure 11:**
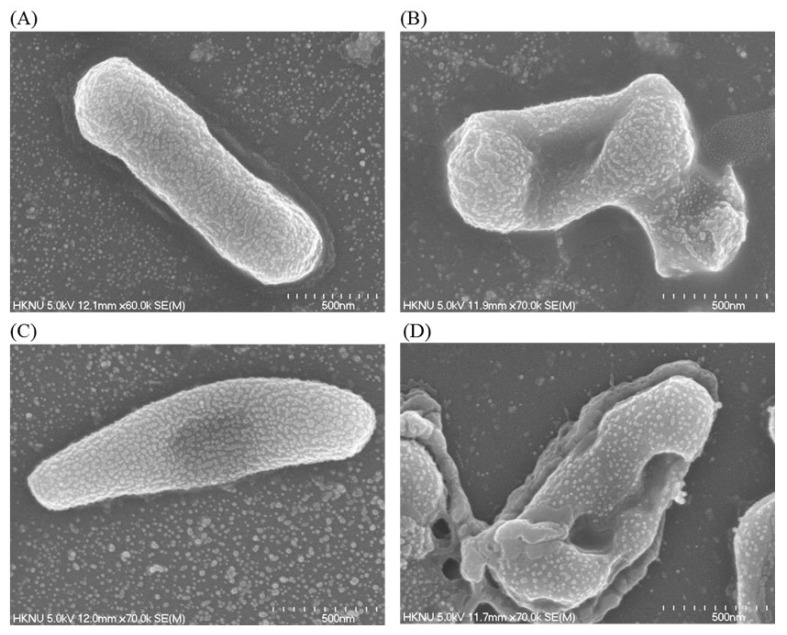
SEM images of normal *E. coli* cells (**A**), 1 × MBC AgNPs treated *E. coli* cells (**B**), normal *P. aeruginosa cells* cells (**C**), and 1 × MBC AgNPs treated *P. aeruginosa cells* cells (**D**).

Structural and morphological changes of *E. coli and P. aeruginosa* cells that were treated by ecofriendly synthesized AgNPs were seen by FE-SEM (field emission scanning electron microscope) (Figure 11). By examining the FE-SEM, the untreated *E. coli* cells were found as a normal rod-shaped and intact cell surface with no damage (Figure 11A). However, *E. coli* cells treated with 1 × MBC of synthesized AgNPs were displayed irregularly wrinkled, damaged, deformed and cracked outer surface, and most of the cell membranes were collapsed (Figure 11B). Similarly, *P. aeruginosa* cells without treatment were observed as the normal rod shape, and the cell surface was intact without any damage (Figure 11C). However, the cells treated with 1×MBC of ecofriendly synthesized AgNPs were displayed as irregularly wrinkled, damaged, deformed and with a cracked outer surface with collapsed cell membranes (Figure 11D). The treated cells depicted a distorted cell structure, indicating the loss of membrane integrity of bacterial cells. The morphological changes, structural alterations and damages of the bacterial cell wall indicated that the ecofriendly synthesized AgNPs might disturb the normal cell functions of both pathogenic strains *E. coli* and *P. aeruginosa,* which would lead to the death of cells. The results of the present study are consistent with previously reported studies [30,31].

## 3. Materials and Methods

### 3.1. Materials

Standard antibiotic discs such as penicillin G (P10) 10 μg/disc, erythromycin (E15) 15 μg/disc and vancomycin (VA30) 30 μg/disc were obtained from Oxoid Ltd., England. Bacterial growth media and Analytical-grade AgNO3 (silver nitrate) were bought from Difco, MB Cell (Seoul, Korea) and Sigma-Aldrich Chemicals (USA), respectively. The pathogenic strains *Escherichia coli* (ATCC 10798) and *Pseudomonas aeruginosa* (ATCC 10145) were collected from American Type Culture Collection (ATCC). 

### 3.2. Isolation of Bacteria

Strain MAHUQ-38^T^ was isolated from the soil sample of a grape garden, located in Anseong, Republic of Korea (37°00′74″ N 127°20′10″ E). The samples were collected in 15 mL conical tubes and suspended in sterile NaCl solution (0.85%, *w*/*v*). Strain MAHUQ-38^T^ was isolated using R2A agar medium through the serial dilution technique after incubation at 30 °C according to the method described by Huq [17]. Then, the isolated strain was sub-cultured on R2A agar and stored at −80 °C in R2A broth with 30% (*v*/*v*) glycerol. Strain MAHUQ-38^T^ has been deposited to Korean Agriculture Culture Collection (KACC) and China General Microbiological Culture Collection Center (CGMCC). 

### 3.3. Phenotypic and Biochemical Characteristics

The Gram reaction was examined according to the previous description [17]. Cell motility was tested using Sulfide-indole-motility (SIM; Difco) medium. Cell morphology was determined by transmission electron microscope, using cells grown on R2A agar (MB-R1129, MB-cell) medium for 2 days at 30 °C. Growth on different media, such as nutrient agar (213000, BD-Difco), R2A agar, Luria–Bertani agar (22700-025, Invitrogen, Carlsbad, CA, USA), MacConkey agar (211387, BD-Difco) and tryptic soy agar (C7121, Criterion), was investigated after 7 days of incubation at 30 °C. Growth at various temperatures (from 5 to 40 °C) and different pH values (from 4 to 10) was checked on R2A agar and R2A broth medium, respectively, after 5 days of incubation at 30 °C. NaCl tolerance test and anaerobic growth ability of strain MAHUQ-38^T^ were conducted according to the previous description [17]. Availability of flexirubin type of pigments were examined according to Sheu et al. [32]. Activities of oxidase, catalase, urease DNase, and hydrolysis of gelatin, casein, starch, Tweens 80 and 20 were investigated according to previous description [17]. Additional biochemical tests were conducted using API 20NE and API ZYM kits, according to the manufacturer’s instructions (bioMérieux). The close type strains, *T. tumescens* KACC 20535^T^, *T. terrae* KACC 11642^T^, *T. terrigena* KCTC 19602^T^, *T. lapilli* LR-26^T^, *T. ginsengihumi* KACC 19444^T^ and *T.* aerolatus KACC 20556^T^ were used as reference strains and were grown under the same experimental conditions of novel strain MAHUQ-38^T^.

### 3.4. 16S rRNA Gene Sequencing and Phylogenetic Analysis

The genomic DNA of strain MAHUQ-38^T^ was extracted and purified using a commercial genomic DNA extraction kit (PDC09-0100, BiO–Helix, Keelung, Taiwan) according to the manufacturer’s instructions. The 16S rRNA gene of strain MAHUQ-38^T^ was amplified from Genomic DNA using bacterial universal primers 27F and 1492R [33], and then the PCR product was purified and sequenced using same primers 27F and 1492R [33] by Solgent Co. Ltd. (Daejeon, Korea). 16S rRNA gene sequences of close type strains were collected from EzBiocloud site (http://www.ezbiocloud.net/eztaxon) [34]. The CLUSTAL_X program was used for multiple sequence alignments [35]. Gaps were edited in the BioEdit program [36]. A Kimura two-parameter model was used to calculate the evolutionary distances [37]. A phylogenic tree was constructed using the neighbor-joining [38] algorithm and MEGA6 program [39] with bootstrap values of 1000 replications [40].

### 3.5. Genome Sequence Analysis

The draft-genome sequence of novel bacterium *T. humi* MAHUQ-38^T^ was analyzed by Illumina HiSeq X Ten and the assembly was completed using de novo assembler (SOAPdenovo v. 3.10.1). The genome annotation was carried out using NCBI prokaryotic genome annotation pipeline (PGAP). The genomic DNA G + C content of strain MAHUQ-38^T^ was directly calculated from its genome sequence. To determine the degree of pairwise relatedness between MAHUQ-38^T^ and close relatives, BLAST-based average nucleotide identity (ANI) was measured according to Yoon et al. [34]. While the digital DNA-DNA hybridization (dDDH) value was evaluated using the genome-to-genome distance calculator (http://ggdc.dsmz.de/ggdc.php) as stated by Meier-Kolthoff et al. [41]. DNA-DNA hybridization was also carried out fluorometrically by the method of Ezaki et al. [42], according to the previous description [43].

### 3.6. Cellular Fatty Acid, Respiratory Quinones and Polar Lipid Analysis

Cellular fatty acid profiles of strain MAHUQ-38^T^ and the reference strains were identified after 48 h of growth on R2A agar medium at 30 °C. The cellular fatty acids were extracted, purified and analyzed according to the previous description [17,44]. The isoprenoid quinones of strain MAHUQ-38^T^ were extracted and purified using the method of Collins and then, analyzed by HPLC [45]. The polar lipids of strain MAHUQ-38^T^ were extracted and analyzed using two-dimensional thin layer chromatography according to the previous description [46].

### 3.7. Ecofriendly Synthesis of AgNPs Using Terrabacter humi sp. nov.

For the synthesis of AgNPs, strain MAHUQ-38^T^ was cultured in 100 mL of R2A broth media for two days at 30 °C using a shaking incubator with 180 rpm. Then, the culture supernatant was separated through centrifuge for 10 min at 10,000 rpm. A total of 100 μL AgNO3 solution (1 M/mL) was mixed with 100 mL of culture supernatant, and then the mixture was incubated at 30 °C in a shaking incubator with dark conditions for 48 h. The biosynthesis of AgNPs was confirmed through visual observation by shifting the color of the culture medium. The biosynthesized AgNPs were collected by centrifugation at 13,500 rpm for 25 min and purified twice through washing by distilled water [13]. Finally, the pellets of AgNPs were air dried and collected for characterization.

### 3.8. Characterization of Synthesized AgNPs

UV–vis spectrophotometer from 300 to 800 nm was used to observe and record the reduction of Ag+ ions. The morphology, size, elemental compositions and SAED (selected area diffraction) pattern of ecofriendly synthesized AgNPs were recorded using FE-TEM (field emission-transmission electron microscopy). A drop of AgNPs solution was placed on the carbon-coated TEM grid, dried at room temperature and finally observed by microscope. Air-dried samples were used for XRD (X-ray diffraction) analysis using an X-ray diffractometer over the 2θ range of 30–90° (D8 Advance, Bruker, Karlsruhe, Germany). The FT-IR (Fourier transform-infrared) spectrum shows the biomolecules which are involved for both the synthesis and stabilization of nanoparticles. The FT-IR spectrum of synthesized AgNPs was recorded by using FTIR spectroscopy in a range of 400–4000 cm^−1^. 

### 3.9. Antimicrobial Activity of Synthesized AgNPs

The antimicrobial activity of ecofriendly synthesized AgNPs against pathogenic microbes was investigated by the Disc diffusion method. The tested pathogenic microbes (*Pseudomonas aeruginosa* and *Escherichia coli*) were grown overnight in MHB (Mueller–Hinton broth). Then, both pathogenic strains (100 μL) were spread completely on an MHA (Mueller–Hinton agar) plate. The sterile paper discs (8 mm) were placed on the surface of the MHA plate, and then 30 μL (both 500 and 1000 ppm) of eco-friendly synthesized AgNPs was given on the paper discs. Finally, the plates were incubated for 24 h at 37 °C. Similarly, the antimicrobial activity of some commercial antibiotics, including vancomycin (30 mg/disc), penicillin G (10 mg/disc) and erythromycin (15 mg/disc), has been evaluated against *P. aeruginosa* and *E. coli*. Inhibition zones were measured after 24 h of incubation. This test was conducted three times.

### 3.10. Determination of Minimal Inhibitory Concentration and Minimal Bactericidal Concentration

The MIC of ecofriendly synthesized AgNPs was also determined by following the broth micro dilution technique. The pathogenic strains *P. aeruginosa* and *E. coli* were grown in MH (Muller–Hinton) broth (70192, Sigma-Aldrich, St. Louis, MO, USA) at 37 °C overnight, and then the optical density was fixed around 1 × 10^6^ CFUs/mL. A total of 100 uL of test bacterial (1 × 10^6^ CFUs/mL) suspension was added into a 96-well ELISA plate followed by an equal volume of AgNPs solution with various concentrations (1.56 to 100 μg/mL), and then the plates were incubated at 37 °C for 24 h. The incubated ELISA plates were measured for absorbance (at 600 nm) using an ELISA plate reader (LabTech 4000). The MBC was determined by streaking of 10 μL of each set on MHA plate and further incubated at 37 °C for 24 h. The culture plates were watched by the naked eye to determine the lowest concentration (MBC) that blocked bacterial growth [47].

### 3.11. Investigation of Structural Changes by FE-SEM

The morphological and structural changes of pathogenic strains *E. coli* and *P. aeruginosa* were examined by FE-SEM. Cells of logarithmic growth phase (1 × 10^7^ CFU/mL) were treated with the biosynthesized AgNPs at the concentration of 1 × MBC. In the control, bacterial cultures were treated with 0.85% NaCl solution. PBS buffer was used to wash the overnight treated cells. At first, the cells were fixed using 2.5% glutaraldehyde and then washed several times using PBS. Once more, the cells were fixed by osmium tetroxide (1%) and subsequently washed with PBS. Fixed cells were dehydrated at room temperature by various concentrations of ethanol (from 30% to 100%) for 10 min. The resulted samples were dried using a desiccator. Finally, the cells were installed on metallic stubs and coated with gold. The structural and morphological changes of the cells were seen by FE-SEM (JSM-7100F, JEOL, Akishima, Tokyo, Japan) [47]. 

## 4. Conclusions

The present study reported the characterization of a novel bacterial species *Terrabacter humi* MAHUQ-38^T^ and also the biologically facile and green synthesis of AgNPs using this novel species and also demonstrated the significant antimicrobial activity of biosynthesized AgNPs against multi-drug-resistant pathogenic microbes. The methodology is ecofriendly, safe, facile, effective and economical. The strain MAHUQ-38^T^ is capable of producing valuable AgNPs within 48 h of incubation. These biosynthesized AgNPs were confirmed by UV–vis spectroscopy, FE-TEM, XRD and FT-IR analysis. Extracellular methodology was used to synthesis AgNPs using *Terrabacter humi* MAHUQ-38^T^ which is simple, green, ecofriendly and cost-effective for large-scale production. Moreover, the biosynthesized AgNPs show significant antimicrobial activity against antibiotic-resistant human pathogenic strain *E. coli* and *P. aeruginosa*. Minimal inhibitory/bactericidal concentration against *E. coli* and *P. aeruginosa* were 6.25/50 and 12.5/50 μg/mL, respectively. The AgNPs altered the cell morphology and damaged the cell membrane of pathogens. Thus, the novel species *Terrabacter humi* MAHUQ-38^T^ could be useful for utilization in the ecofriendly synthesis of AgNPs for various applications in both medical and non-medical sectors, especially as an antimicrobial agent to control drug-resistant pathogens.

## Figures and Tables

**Table 1:** Differential characteristics of *Terrabacter humi* MAHUQ-38^T^ and phylogenetically closely related *Terrabacter* species. Strains: 1, *T. humi* MAHUQ-38^T^; 2, *T. tumescens* KACC 20535^T^; 3, *T. terrae* KACC 11642^T^; 4, *T. terrigena* KCTC 19602^T^; 5, *T. lapilli* LR-26^T^; 6, *T. ginsengihumi* KACC 19444^T^ and 7, *T.* aerolatus KACC 20556^T^.

Characteristics	1	2	3	4	5	6	7
Isolation source	Soil	Soil	Soil	Soil	Stone	Soil	Air
Cell morphology	Rod	Rod/coccus cycle	Long rods	Short rods/rods	Short rods	coccus-rod	Rod/coccoid
Colony color	Milky white	White/grey	Yellow	Greyish yellow	Bright yellow	yellowish	White
Motility	-	-	-	-	-	-	+
Reduction of nitrate (API 20 NE)	+	+	+	-	+	+	+
Arginine dihydrolase	-	-	+	-	-	-	-
Growth temperature (°C)	10–40	10–35 ^a^	15–40 ^b^	10–37 ^c^	10–40 ^d^	18–37 ^e^	5–35 ^f^
NaCl tolerance (%)	0–5	0–5 ^a^	0–7 ^b^	0–3 ^c^	0–3 ^d^	0–5 ^e^	0–5 ^f^
**Hydrolysis of:**							
Casein	+	+	+	+	+	-	+
Starch	-	-	+	-	+	-	W
DNA	+	+	-	W	+	-	-
l-Tyrosine	+	-	-	-	-	W	+
Urea (API 20 NE)	-	-	+	-	-	-	-
**Enzyme activity (API ZYM):**							
Esterase (C4)	+	+	+	+	+	-	+
Alkaline phosphatase	+	-	-	+	+	-	-
Esterase lipase (C8)	+	+	+	+	+	-	+
Lipase (C14)	W	-	+	+	+	+	+
Valine arylamidase	+	-	+	+	+	-	+
Cystine arylamidase	+	-	-	+	+	-	-
Trypsin	W	-	-	-	+	-	-
α-chymotrypsin	-	-	+	-	+	-	-
β-glucuronidase	-	-	+	-	+	+	-
α-mannosidase	-	-	+	+	-	-	W
*a*-glucosidase	+	-	+	+	+	+	+
*a*-galactosidase	+	-	+	+	+	+	+
β-glucosidase	W	-	+	+	+	+	W
β-galactosidase	+	-	+	+	+	+	+
**Assimilation of (API 20 NE):**							
l-arabinose	W	+	-	-	-	-	-
*N*-acetyl-glucosamine	+	+	+	+	+	-	+
d-maltose	+	-	-	+	+	-	+
d-mannose	+	+	+	+	-	+	+
d-mannitol	-	+	-	+	-	+	+
DNA G + C content (mol%)	70.8	69.2–72.4 ^a^	71.0 ^b^	71.6 ^c^	72.6 ^d^	70.5 ^e^	71.7 ^f^

All data were obtained in this study, except ^a–e^ and ^f^ that were taken from Collins et al. [1], Montero-Barrientos et al. [4], Yoon et al. [6], Lee et al. [5], Jin et al. [3], Weon et al. [2], respectively. All strains are aerobic. All strains are positive for catalase, esculin, gelatin, leucine arylamidase and naphthol-AS-BI-phosphohydrolase, assimilation of d-glucose and malic acid. All strains are negative for oxidase, indole production, glucose fermentation, N-acetyl-β-glucosaminidase and α-fucosidase, assimilation of adipic acid, capric acid and triosodium citrate. API, analytical profile index; +, Positive; W+, weakly positive; −, negative.

**Table 2:** Genome sequence features of *Terrabacter humi* MAHUQ-38^T^.

Features	Strain MAHUQ-38^T^
Accession No.	JACVCU000000000
Biosample	SAMN15891656
BioProject	PRJNA658849
Total sequence length (nt)	5,156,829
Scaffold N50	910,522
Scaffold L50	3
Number of contigs	19
Sequencing method	de novo
Annotation pipeline	NCBI Prokaryotic Genome
DNA G + C content (mol%)	70.8
Total genes	4664
Genes (coding)	4555
Number of RNAs	56
tRNAs	48
rRNAs	5

**Table 3:** Cellular fatty acid composition of *Terrabacter humi* MAHUQ-38^T^ and phylogenetically closely related *Terrabacter* species. Strains: 1, *T. humi* MAHUQ-38^T^; 2, *T. tumescens* KACC 20535^T^; 3, *T. terrae* KACC 11642^T^; 4, *T. terrigena* KCTC 19602^T^; 5, *T. lapilli* LR-26^T^; 6, *T. ginsengihumi* KACC 19444^T^ and 7, *T.* aerolatus KACC 20556^T^.

Fatty Acid	1	2	3	4	5	6	7
C_13:0 iso_	-	-	9.3	2.9	10.1	-	Tr
C_14:0_	-	8.4	2.4	5.7	-	Tr	Tr
C_14:0 iso_	24.6	4.5	29.1	15.6	32.8	14.7	9.8
C_14:1_ ω5c	1.5	5.5	-	-	-	-	-
C_15:0 iso_	24.4	6.0	-	11.2	-	34.2	42.8
C_15:0 anteiso_	12.3	2.1	Tr	-	-	8.2	7.2
C_15:1 iso_ F	-	-	23.9	3.3	19.6	-	-
C_15:1_ ω6c	1.4	-	-		-	-	-
C_16:0_	-	13.9	3.5	15.1	1.8	Tr	3.7
C_16:0 iso_	16.8	3.4	4.7	--	3.1	21.3	15.1
C_16:0_ 3-OH	-	-	-	7.1	-	-	-
C_16:1_ 2-OH	-	-	4.8	-	-	-	-
C_16:1 iso_ H	5.0	-	-	5.5	2.2	6.4	1.0
C_17:0_	-	6.3	2.8	-	5.4	-	1.6
C_17:0 iso_	-	-	-	-	-	Tr	4.7
C_17:0 anteiso_	1.7	-	-	-	Tr	1.2	3.5
C_17:0_ 10-methyl	-	2.3	-	-	-	1.0	Tr
C_17:0_ cyclo	-	-	3.1	4.7	1.6	-	-
C_17:1_ ω8c	4.6	11.9	2.6	-	3.2	1.9	1.2
C_17:1 anteiso_ ω9c	1.3	-	-	-	-	1.1	Tr
C_17:1 anteiso_ A	-	-	1.3	1.5	2.1	-	-
C_18:0_	-	3.7	1.2	4.7	Tr	-	3.4
C_18:0 iso_	-	-	4.5	10.4	6.8	-	1.1
C_18:1_ ω9c	-	16.5	-	6.2	Tr	1.5	1.0
C_19:0 iso_	-	-	2.2	-	3.5	-	-
Sum In Feature 3	1.3	6.5	-	-	-	-	1.4
Sum In Feature 9	4.4	8.0	-	-	-	-	1.3

Sum in Feature 3 = C_16:1_ w7c and/or C_16:1_ w6c. Sum in Feature 9 = C_17:1 iso_ ω9c and/or C_16:0_ 10-methyl. All data were collected from this study. Tr, trace (less than 1%); -, not detected.

**Table 4:** Antimicrobial activity of eco-friendly synthesized AgNPs against *E. coli* and *P. aeruginosa*.

Pathogenic Species	Zone of Inhibition (mm)	
	1000 ppm	500 ppm
*Escherichia coli* [ATCC 10798]	15.6 ± 1.0	13.8 ± 0.7
*Pseudomonas aeruginosa* [ATCC 10145]	14.1 ± 1.2	13.4 ± 0.8

**Table 5:** Antimicrobial activity of commercial antibiotics against *E. coli* and *P. aeruginosa*.

Pathogenic Species	Antibiotic	Zone of Inhibition (mm)
*Escherichia coli* [ATCC 10798]	Erythromycin	-
	Vancomycin	-
	Penicillin G	-
*Pseudomonas aeruginosa* [ATCC 10145]	Erythromycin	*-*
	Vancomycin	*-*
	Penicillin G	*-*

## Supplementary Materials

Supplementary materials can be found at https://www.mdpi.com/1422-0067/21/24/9746/s1.
