# Supplementary material for: Ecofriendly Synthesis of Silver Nanoparticles by Terrabacter humi sp. nov. and Their Antibacterial Application against Antibiotic-Resistant Pathogens
Source: Int J Mol Sci. 2020 Dec 21;21(24):9746. doi: 10.3390/ijms21249746 (PMC7766514; doi:10.3390/ijms21249746)
Supplement: Supplementary file 1 [file ijms-21-09746-s001.pdf]

Supplementary Materials

Ecofriendly Synthesis of Silver Nanoparticles by *Terrabacter humi* sp. nov. and Their Antibacterial Application against Antibiotic-Resistant Pathogens.

Shahina Akter[1], Sun-Young Lee[2], Muhammad Zubair Siddiqi[3], Sri Renukadevi Balusamy[4], Ashrafudoulla[5], Esrat Jahan Rupa[6], Md. Amdadul Huq[2*]

[1] *Department of Food Science and Biotechnology, Gachon University, Seongnam, 461-701, Republic of Korea.*

[2] *Department of Food and Nutrition, Chung-Ang University, Anseong, Gyeonggi-do, 17546, Republic of Korea*

[3] *Department of Biotechnology, Hankyong National University, Anseong, Gyeonggi-do, 17579, Republic of Korea*

[4] *Department of Food Science and Technology, Sejong University, Gwangjin-gu, Seoul, 143-747, Republic of Korea*

[5] *Department of Food Science and Technology, Chung-Ang University, Anseong-si, Gyeonggi-do 17546, Korea;*

[6] *Dept.of oriental medicinal material processing. Kyung Hee university, Yongin-si, Republic of Korea.*

[*] **Corresponding author:**

Md. Amdadul Huq,

Chung-Ang University, Anseong-si, Gyeonggi-do, 17546, Republic of Korea.

Tel: +82-031-670-4568

E-mail: amdadbge@gmail.com ; amdadbge100@cau.ac.kr

Supplementary Table S1. dDDH and ANI values between proposed novel species *Terrabacter humi* MAHUQ-38^T and the close type strains of the genus *Ramlibacter*.

Query genome	Reference genome	Formula 1				Formula 2				Formula 3				ANI (%)
		DDH	Model C.I.	Distance	Prob. DDH >= 70%	DDH	Model C.I.	Distanc e	Prob. DDH >= 70%	DDH	Model C.I.	Distanc e	Prob. DDH >= 70%	
Terrabacter soli MAHUQ-38^T (JACVCU000000000)	*T. tumescens* JCM 1365^T (GCM10009721)	40.30	[36.9 - 43.7%]	0.3899	1.8	27.20	[24.8 - 29.7%]	0.1590	0.03	36.00	[33.1 - 39.1%]	0.4869	0.03	83.71
	T. aerolatus NBRC 106305^T (GCA_007991735.1)	38.70	[35.3 - 42.2%]	0.4055	1.23	26.70	[24.4 - 29.2%]	0.1618	0.02	34.80	[31.8 - 37.8%]	0.5017	0.02	83.44

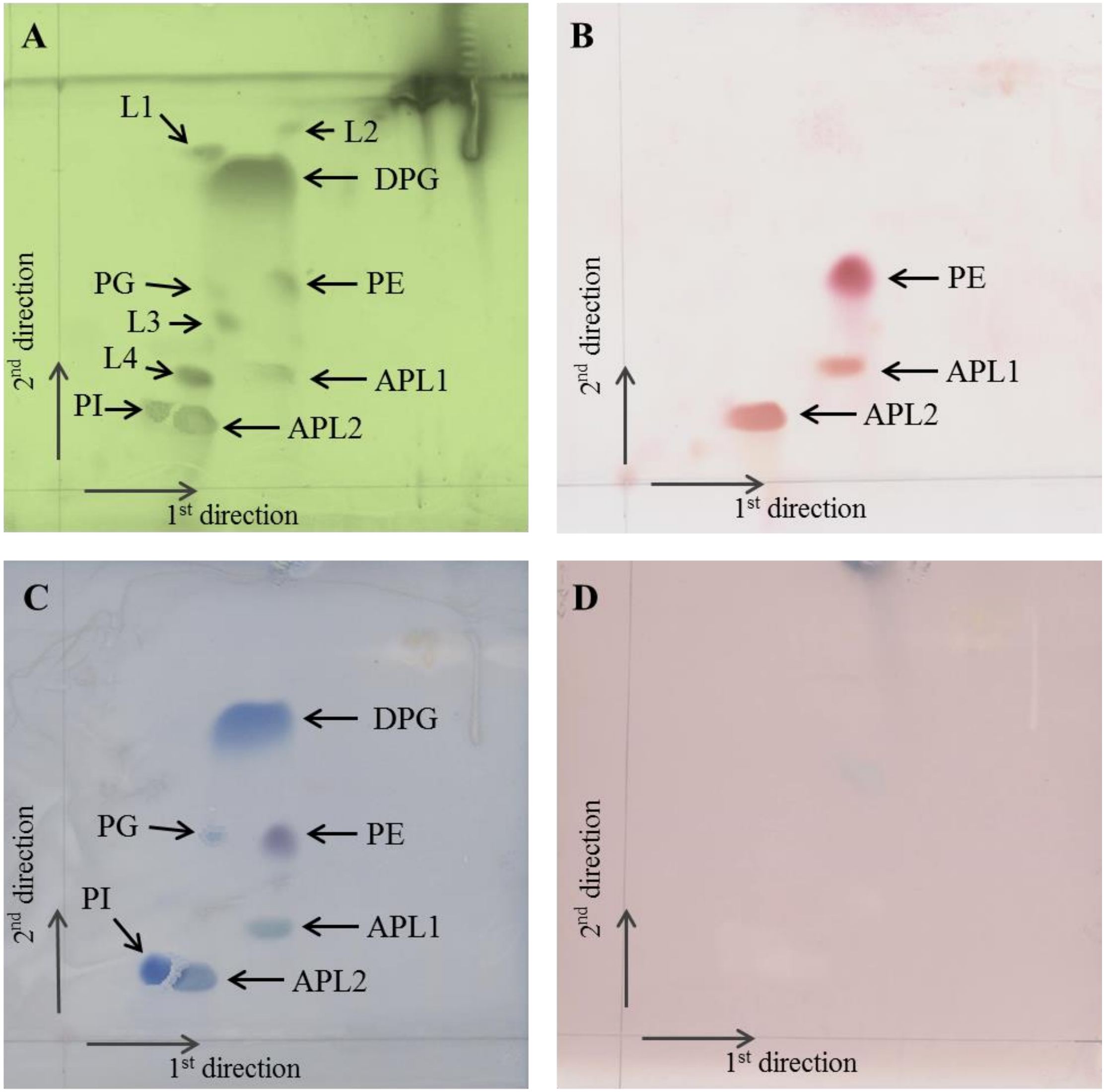

Supplementary Figure S1. Two-dimensional TLC of the polar lipids of *Terrabacter humi* MAHUQ-38^T. (A) Total lipids detective by spraying with 5% ethanolic molybdophosphoric acid; (B) Aminolipids detected by spraying with 0.2% (w/v) ninhydrin; (C) Phospholipids detected by spraying with molybdenum blue; and (D) Glycolipids were analysed by α naphthol reagent. Abbreviations: DPG, diphosphatidylglycerol; PG, phosphatidylglycerol; PE, phosphatidylethanolamine; PI, phosphatidylinositol; APL1-2, unidentified aminophospholipids; and L1-4, unidentified polar lipids.
